# Purine signaling pathway dysfunction in autism spectrum disorders: Evidence from multiple omics data

**DOI:** 10.3389/fnmol.2023.1089871

**Published:** 2023-02-03

**Authors:** Si Dai, Jingjing Lin, Yanting Hou, Xuerong Luo, Yidong Shen, Jianjun Ou

**Affiliations:** Department of Psychiatry, National Clinical Research Center for Mental Disorders, The Second Xiangya Hospital of Central South University, Changsha, Hunan, China

**Keywords:** autism spectrum disorders (ASD), purine metabolism, metabolomics (OMICS), transcriptomics, RNA sequencing (RNA-seq), uric acid (UA)

## Abstract

**Introduction:**

Previous studies have suggested that the dysregulation of purine metabolism may be associated with autism spectrum disorder (ASD). Here, we adopted metabolomics and transcriptomics to verify and explore the underlying molecular mechanism of purine metabolism dysfunction in ASD and identify potential biomarkers within the purine metabolism pathway.

**Methods:**

Ultra-high-performance liquid chromatography-mass spectrometry was used to obtain the plasma metabolic profiles of 12 patients with ASD and 12 typically developing (TD) children. RNA sequencing was used to screen differentially expressed genes related to the purine metabolic pathway and purine receptor-coding genes in 24 children with ASD and 21 healthy controls. Finally, serum uric acid levels were compared in 80 patients with ASD and 174 TD children to validate the omics results.

**Results:**

A total of 66 identified metabolites showed significant between-group differences. Network analysis showed that purine metabolism was the most strongly enriched. Uric acid was one of the most highlighted nodes within the network. The transcriptomic study revealed significant differential expression of three purine metabolism-related genes (adenosine deaminase, adenylosuccinate lyase, and bifunctional enzyme neoformans 5-aminoimidazole-4-carboxamide ribonucleotide (AICAR) transformylase/inosine monophosphate (IMP) cyclohydrolase) (*p* < 0.01) and five purinergic receptor genes (P2X7, P2Y2, P2Y6, P2Y8, and P2Y10) (*p* < 0.05). In the validation sample, there was a significant difference in serum uric acid levels between the two groups (*p* < 0.001), and the area under the curve for uric acid was 0.812 (sensitivity, 82.5%; specificity, 63.8%).

**Discussion:**

Patients with ASD had dysfunctional purine metabolic pathways, and blood uric acid may be a potential biomarker for ASD.

## 1. Introduction

Autism spectrum disorder (ASD) is a neurodevelopmental disorder characterized by impairments in social interaction, stereotyped behavioral patterns, and narrow interests (Francesmonneris et al., 2013). The global prevalence of ASD has increased dramatically over the past few decades, and to date, the prevalence of the disease has reached 1 in 44, with its prevalence being 4.2 times higher in boys than in girls (Maenner et al., 2021). Currently, the etiology of ASD is unknown, with its occurrence and progression being influenced by genetic and environmental risk factors (Taylor et al., 2020). Several pathophysiological mechanisms have been found to play a key role in the development of ASD, such as oxidative stress, neuroinflammation, immune dysregulation, and mitochondrial dysfunction (Vargas et al., 2005; Saffari et al., 2019; Citrigno et al., 2020; Chen et al., 2021).

The diagnostic tools for ASD are limited, and the diagnosis is mainly based on scale assessment; however, objective biological diagnostic indicators are lacking. A previous study shows that most ASD is not diagnosed in children until they are 4 years of age or older, which may lead to a delay in treatment (Christensen et al., 2019). With the current lack of medications for core ASD symptoms, special education training remains the mainstay to improve patient function, and the earlier it is performed, the better (Howes et al., 2018). Therefore, to better target the underlying causes of ASD for diagnosis and treatment, it is essential to identify reliable biomarkers closely related to ASD (Shen et al., 2020). Biomarkers help not only in early objective diagnosis but also in identifying the pathophysiological mechanisms that influence the development of the disease, thus allowing therapeutic interventions to stop or improve the development of specific pathologies before the onset of obvious psychiatric and behavioral symptoms (Marin, 2016).

Multiple metabolomics studies have identified dysregulated purine metabolism in children with ASD, as well as abnormalities in multiple metabolites in the purine metabolic pathway, such as uric acid, inosine, and purine products (Gevi et al., 2016; Bitar et al., 2018; Kurochkin et al., 2019; Xiong et al., 2019; Liang et al., 2020; Mussap et al., 2020). It is worth noting that uric acid, an end product of the purine metabolic pathway, is a hydrophilic antioxidant that exerts neuroprotective effects through antioxidative stress (Glantzounis et al., 2005; Bowman et al., 2010). In addition, abnormal metabolism of enzymes related to purine metabolism is associated with ASD. Adenylosuccinate lyase (ADSL) is a key enzyme in purine *de novo* synthesis and purine nucleotide recycling pathways, and ADSL deficiency may lead to ASD symptoms in some patients (Jaeken et al., 1988; Toth and Yeates, 2000). However, the evidence is limited and not always consistent, which may partly be due to the small sample size or differences in the tissues tested. Other sources of inconsistency include differences in daily living, such as dietary habits, which may vary in different regions or ethnic groups (Sanctuary et al., 2018). Hence, it is necessary to replicate and verify previous findings in a particular population.

Moreover, the relationship between abnormal purine metabolism and the etiology of ASD is currently unclear, and further research is necessary to investigate the molecular or genetic origin of purine pathway abnormalities. Previous studies have indicated that purinergic signaling may be involved in neurodevelopmental processes such as cell proliferation, differentiation, and neuron-glial cell interactions (Burnstock et al., 2011). Depending on the ligand, purinergic signaling receptors are divided into two major classes: P1 (adenosine receptors) and P2 (ATP/ADP and UTP/UDP receptors) (Burnstock, 2007). The latter includes P2X and P2Y, which mediate glial cell hyperactivation and the onset of inflammatory responses in the central nervous system (CNS) (Abbracchio and Ceruti, 2006; Inoue, 2008; Huang et al., 2019).

It is known that omics is an effective method for finding biomarkers (Shen et al., 2020). Transcripts of an organism can reflect the dynamics of changes in the genes being expressed, while metabolites can directly and accurately reflect the pathophysiological state of the organism. Therefore, this study used a combined multi-omics analysis to identify diagnostic biomarkers within the purine metabolism pathway more comprehensively.

## 2. Materials and methods

### 2.1. Participants

This study contains three sample sets: metabolome, transcriptome, and validation sets, which were used for ultra-high-performance liquid chromatography mass spectrometry (UHPLC-MS/MS) analysis, RNA sequencing (RNA-seq), and blood uric acid detection, respectively. During 2018–2019, all ASD patients were recruited from the Second Xiangya Hospital of Central South University (Changsha, China), and typically developing (TD) children as healthy controls were recruited from routine physical examinations in general schools in Changsha, China.

The enrollment criteria for all ASD children were as follows: (1) age 3–16 years; (2) met the American Diagnostic and Statistical Manual of Mental Disorders-IV Text Revision (DSM-IV-TR) diagnostic criteria for ASD; and (3) met the classification of ASD by the validated Chinese version of the Autism Diagnostic Interview-Revised (ADI-R) and the Autism Diagnostic Observation Schedule (ADOS). The exclusion criteria for ASD were as follows: (1) serious physical diseases or disabilities, such as congenital heart disease, thyroid disease, diseases with severely abnormal liver or kidney function, and diseases with abnormal vision or hearing; (2) serious neurological disorders, such as traumatic brain injury, encephalitis, epilepsy, hyperthermia, birth injury, and electroencephalography (EEG) abnormalities; (3) known history of genetic disorders or syndromes, such as Down syndrome and Fragile-X syndrome; (4) other neurodevelopmental disorders, such as attention-deficit/hyperactivity disorder, specific learning disorders, and motor disorders; and (5) other severe mental illnesses, such as schizophrenia and bipolar disorder. The enrollment criteria for healthy control included: (1) age 3–16 years; and (2) typically developing children without obvious language, behavioral, and social problems. The exclusion criteria for healthy controls were consistent with those of the ASD group.

The study was approved by the Ethics Committee of the Second Xiangya Hospital of Central South University, with voluntary participation as the principle, to sign an informed consent form after all parents or other legal guardians were informed about the relevant content of this study in detail.

### 2.2. Blood sample collection

#### 2.2.1. Metabolome set

Fasting peripheral blood from children with ASD and TD was collected in 5 ml using EDTA anticoagulation tubes, mixed thoroughly, and then centrifuged for 10 min. After centrifuging at 2000 rpm for 10 min at 4°C using a cryogenic centrifuge, the plasma (supernatant) was dispensed into multiple 0.5-ml centrifuge tubes and stored in a −80°C refrigerator for uniform testing.

#### 2.2.2. Transcriptome set

Venous blood (10 ml) was collected from the peripheral vein of the children using a whole blood RNA tube (BD PAXgene tube), which was then gently inverted 8–10 times after collection to mix the protective agent in the tube with the blood more thoroughly, and stored in a −80°C refrigerator.

#### 2.2.3. Validation set

Fasting peripheral blood (2 ml) was collected from children using separated gel coagulation-promoting tubes, stored at room temperature, and protected from light for half an hour after collection. The supernatant was aspirated after centrifugation at 3000 rpm for 10 min to obtain serum. Serum samples were sent to a fully automated biochemical analyzer (7170A, Japan) for detection of uric acid concentration within 2 h after collection.

### 2.3. UHPLC-MS/MS analysis

LC-MS/MS analyses were performed using the Vanquish UHPLC system (Thermo Fisher Scientific, Waltham, MA, USA) coupled with an Orbitrap Q Exactive HF-X mass spectrometer (Thermo Fisher Scientific, Waltham, MA, USA). Q Exactive HF-X mass spectrometer was operated in positive/negative polarity mode with a spray voltage of 3.2 kV, a capillary temperature of 320°C, a sheath gas flow rate of 35 arb, and an auxiliary gas flow rate of 10 arb (see Supplementary methods 1 for details).

### 2.4. RNA-seq

RNA integrity was assessed using the RNA Nano 6000 Assay Kit and the Bioanalyzer 2100 software (Agilent Technologies, Palo Alto, CA, USA). Libraries were constructed according to the instructions of the NEBNext^®^ UltraTM RNA Library Prep Kit (New England Biolabs Inc., Ipswich, MA, USA). The effective concentration of the library was quantified using qRT-PCR to ensure the quality of the library.

The basic principle of sequencing is sequencing during synthesis. Clustering of index-coded samples was performed on a cBot Cluster Generation System using the TruSeq PE Cluster Kit v3-cBot-HS (Illumina, San Diego, CA, USA). After cluster generation, the library preparations were sequenced on an Illumina Novaseq platform, and 150 bp paired-end reads were generated (see Supplementary methods 2 for details).

### 2.5. Data processing and statistical analysis

#### 2.5.1. Metabolomic analysis

The raw data files generated by UHPLC-MS/MS were processed using Compound Discoverer 3.0 (CD 3.0, Thermo Fisher) to perform peak alignment, peak picking, and quantitation for each metabolite. The main parameters were set as follows: retention time tolerance, 0.2 min; actual mass tolerance, 5 ppm; signal intensity tolerance, 30%; signal/noise ratio, 3; and minimum intensity, 100000. Peak intensities were normalized to the total spectral intensity. The peaks were then matched using the mzCloud^1^ and ChemSpider^2^ databases to obtain accurate qualitative and quantitative results.

A comprehensive analysis of the processed metabolomics dataset was performed using the MetaboAnalyst 5.0^3^ web tool (Pang et al., 2021). The univariate analysis included Student’s *t*-test and fold change (FC) analysis. To correct for multiple testing and false positives, we used a false discovery rate (FDR) cut-off of *P* < 0.05. For multivariate statistical analysis, orthogonal partial least squares discriminant analysis (OPLS-DA) was performed, and the variable importance in the projection (VIP) value of each variable was calculated. VIP > 1, *p* < 0.05, and FDR < 0.05 were used to screen for significantly differential metabolites. These metabolites were subsequently subjected to network analysis (see Supplementary methods 3 for details). Receiver operating characteristic (ROC) curves and area under the curve (AUC) were calculated using SPSS26 to quantify the diagnostic performance of the differential metabolites.

#### 2.5.2. Transcriptome analysis

Sequenced fragments were converted into sequence data (reads) by CASAVA base identification, which mainly includes the sequence information and quality information of the fragment. Through the data quality control steps of raw data filtering, as well as inspections of sequencing error rate and GC content distribution, sequence data used for subsequent analysis (clean reads) were obtained. Annotation files for the reference genome and gene model were downloaded directly from the genome website. The index of the reference genome was built using Hisat2 v2.0.5, and clean paired-end reads were aligned to the reference genome using Hisat2 v2.0.5. FeatureCounts v1.5.0-p3 was used to count the number of reads mapped to each gene.

Using the Kyoto Encyclopedia of Genes and Genomes (KEGG) pathway annotation, the following genes related to the purine metabolism pathway were identified: (1) enzymes for purine synthesis, recycling, and metabolism, including phosphoribosyl pyrophosphate synthase (PRPS), adenosine deaminase (ADA), adenylosuccinate lyase (ADSL), the bifunctional enzyme neoformans 5-aminoimidazole-4-carboxamide ribonucleotide (AICAR) transformylase/inosine monophosphate (IMP) cyclohydrolase (ATIC), and hypoxanthine guanine phosphoribosyltransferase (HPRT); (2) purinergic receptors, divided into P1 and P2 receptors, where P1 receptors include A1, A2A, A2B, and A3 receptors; P2 receptors include P2X1, P2X4, P2X5, P2X6, P2X7, P2Y1, P2Y2, P2Y4, P2Y6, P2Y8, P2Y10, P2Y11, P2Y12, P2Y13, and P2Y14.

Differential expression analysis of these genes was performed using the DESeq2 R package (1.20.0). The resulting *P*-values were adjusted using Benjamini and Hochberg’s approach for controlling the FDR. Adjusted *p*-values < 0.05 and absolute FC > 1.5 (| log2FC| > 0.585) were set as the threshold for significantly differential gene expression.

#### 2.5.3. Validation set analysis

Using SPSS26, the Mann–Whitney *U* test was used to compare blood uric acid concentrations between the ASD and TD groups. Binary logistic regression and ROC curve analysis were used to determine the adjunctive diagnostic value of uric acid in children with ASD.

## 3. Results

### 3.1. Demographic and clinical characteristics of participants

**FIGURE 1:**
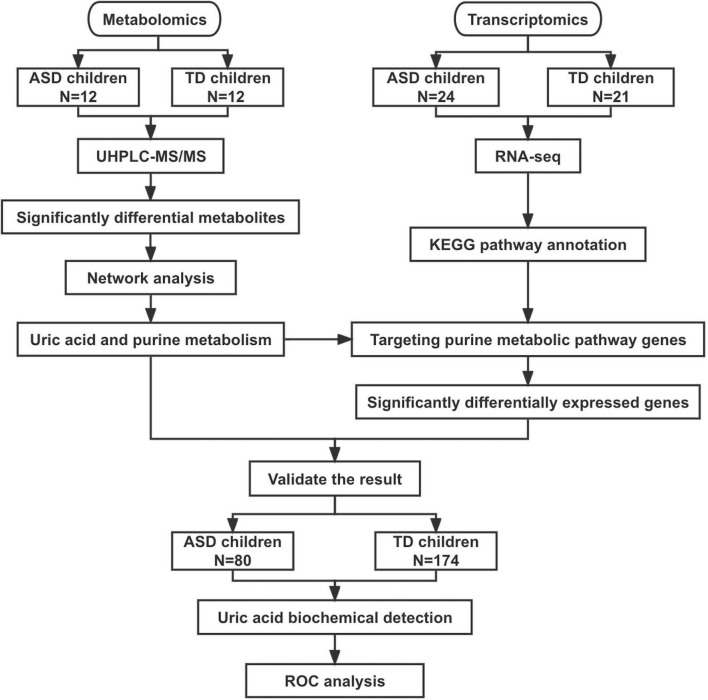
Flowchart of this study. ASD, autism spectrum disorder; TD, typically developing; UHPLC-MS/MS, ultra-high-performance liquid chromatography mass spectrometry; ROC, receiver operating characteristic curve.

Three datasets were included in this study (Figure 1). The metabolome set included plasma samples from 24 boys (12 ASD patients aged 9–13 years and 12 TD children aged 10–14 years) used for metabolomic analysis. The transcriptome set included blood samples from 24 ASD patients (male/female: 24/0, 10–15 years) and 21 TD children (male/female: 10/11, 13–14 years) used for transcriptome analysis. The validation set included 80 patients with ASD (male/female: 75/5, 3–16 years) and 174 children with TD (male/female: 152/22, 3–16 years). The clinical and demographic characteristics of the participants are shown in Table 1.

**TABLE 1:** Demographic characteristics of the study participants.

Characteristics	Metabolomics sample set		Transcriptome sample set		Validation sample set	
	ASD (*n* = 12)	TD (*n* = 12)	ASD (*n* = 24)	TD (*n* = 21)	ASD (*n* = 80)	TD (*n* = 174)
Male, n (%)	12 (100.0)	12 (100.0)	24 (100.0)	10 (47.6)	75 (93.8)	152 (87.4)
Age (years, Mean ± SD)	11.00 ± 1.54	13.08 ± 1.31	11.38 ± 1.56	13.62 ± 0.50	9.03 ± 3.49	9.11 ± 3.44
**ADOS (Mean ± SD)**						
Communication	6.82 ± 2.23	—	7.63 ± 2.34	—	7.53 ± 2.13	—
Reciprocal social interaction	10.35 ± 2.01	—	10.33 ± 2.85	—	10.92 ± 2.28	—
Communication + social interaction	17.18 ± 3.82	—	17.96 ± 4.97	—	18.45 ± 4.13	—
Play/Imagination and Creativity	2.64 ± 1.29	—	3.00 ± 1.22	—	2.91 ± 1.17	—
Stereotyped behavior and restricted interests	2.09 ± 2.34	—	3.29 ± 2.65	—	3.24 ± 2.13	—
**ADI-R (Mean ± SD)**						
Qualitative abnormalities in social interaction	24.00 ± 4.57	—	26.38 ± 3.70	—	23.99 ± 4.41	—
Qualitative abnormalities in communication	18.00 ± 4.82	—	18.75 ± 3.59	—	17.64 ± 4.24	—
Restricted, repetitive, and stereotyped patterns of behavior	6.75 ± 3.82	—	6.83 ± 2.75	—	6.08 ± 2.65	—
Abnormality of development evident at or before 36 months	3.25 ± 1.06	—	3.75 ± 1.19	—	3.76 ± 1.32	—

ADOS, autism diagnostic observation schedule; ADI-R, autism diagnostic interview-revised.

### 3.2. Metabolite identification

Twenty-four plasma samples were subjected to untargeted metabolomics analysis, which identified 611 positive-mode features and 170 negative-mode features. After log transformation and Pareto scaling of the data, a *t*-test, fold-change analysis, and OPLS-DA analysis were performed. As shown in Supplementary Figure 1, there were remarkable separations between ASDs and TDs. After the permutation test, all models were statistically significant (*p* ≤ 0.001). Finally, 66 identified metabolites showed significant group differences (VIP > 1 and FDR-P < 0.05) (Supplementary material).

**FIGURE 2:**
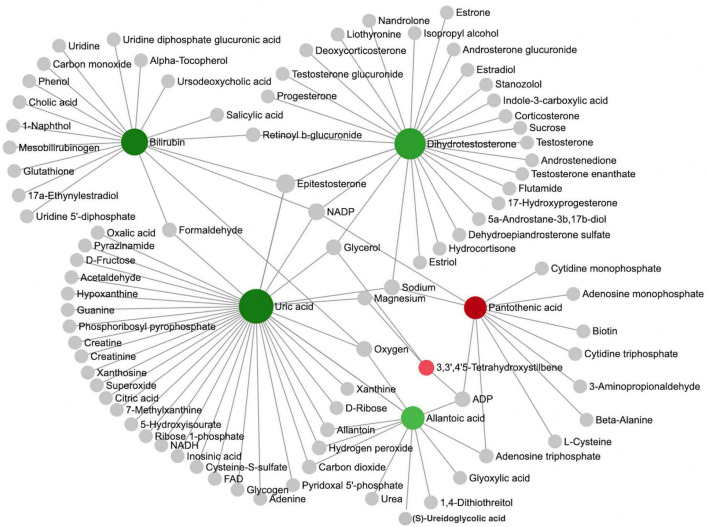
Analysis of metabolic networks between significantly different metabolites. The chemical-chemical associations of the metabolite networks were extracted from STITCH (http://stitch.embl.de/), using only highly confident interactions. In graph networks, nodes with a higher number of connections to other nodes are more important and have larger node sizes, and they act as hubs in the network. The colors represent the direction of change of ASD relative to the TD group: green nodes indicate a decrease, and red nodes indicate an increase. The metabolites represented by the gray nodes are not included in the results found in this study but are based on metabolites associated with similar chemical structures and similar molecular activities.

We further performed a metabolite-metabolite network analysis of the differential metabolites to highlight the potential functional relationships among them. Uric acid, dihydrotestosterone (androstanolone), bilirubin, pantothenic acid, allantoic acid, and 3,3’,4’5-tetrahydroxystilbene (piceatannol) were the most highlighted nodes in the network (Figure 2). Concomitantly, this network analysis module also performed a KEGG enrichment analysis of these significant metabolites, which revealed that purine metabolism and steroid hormone biosynthesis were most strongly enriched (Supplementary Table 1). Details of the significant metabolites are presented in Table 2. Compared with TDs, uric acid, allantoic acid, bilirubin, and dihydrotestosterone (androstanolone) levels decreased, while D-pantothenic acid and 3,3’,4’5-tetrahydroxystilbene (piceatannol) levels increased in ASD patients.

**TABLE 2:** Significant differential metabolites and their metabolic pathways derived from network analysis discriminated between ASD and TD children.

Metabolite	FDR-P value	VIP	Fold change	Trend^a^	Pathway
Uric acid	0.001	2.42	0.48	↓	Purine metabolism
Dihydrotestosterone	0.026	1.90	0.60	↓	Steroid hormone biosynthesis
Allantoic acid	0.040	1.71	0.71	↓	Purine metabolism
Bilirubin	0.046	1.77	0.48	↓	Porphyrin and chlorophyll metabolism
D-Pantothenic acid	0.040	1.76	1.70	↑	Pantothenate and CoA biosynthesis
3,3’,4’5-tetrahydroxystilbene	0.002	2.17	1.16	↑	Stilbenoid, diarylheptanoid, and gingerol biosynthesis

FDR-P values, adjusted *P*-values by False Discovery Rate method; VIP, variable importance in the projection.

^a^The arrow shows the change direction of ASD group relative to TD group: ↑ means increase, ↓ means decrease.

To quantify the diagnostic performance of these six plasma metabolites, a ROC curve analysis was performed. The results showed that uric acid had the highest AUC, with a value of 0.958 (*p* < 0.001). Therefore, we performed validation of uric acid in a larger sample. The ROC curves of other metabolites ranked by AUC are shown in Supplementary Table 2.

### 3.3. Transcriptomics result

Forty-five samples were subjected to RNA transcriptional sequencing to obtain read counts of individual gene expression for each sample. The results showed significant differences in the expression of purine metabolism-related genes ADA, ADSL, and ATIC between ASDs and TDs, in which, ADA and ATIC were significantly up-regulated while ADSL was significantly down-regulated (Table 3; Supplementary Table 3). The normalized count values of purinergic receptor transcripts in ASDs and TDs were measured using the same method as above. The results showed significant differences in the expression of P2X7, P2Y2, P2Y6, P2Y8, and P2Y10, with the expression of P2Y2, P2Y6, and P2Y8 being significantly upregulated, while the expression of P2X7 and P2Y10 being significantly down-regulated (Table 3; Supplementary Table 3). The results of the ROC curve analysis of purine metabolism-related genes are shown in Supplementary Table 2.

**TABLE 3:** Significant differentially expressed genes and their trends were obtained by comparing the normalized counts of gene expression in the ASD and TD groups.

Gene name	ASD (*n* = 24)		TD (*n* = 21)		Between-group difference		
	Mean	SD	Mean	SD	*P*-adj^a^	Log2FC	Trend^b^
**Purine pyrimidine synthase**							
ADA	456.09	79.24	353.47	59.63	<0.001	0.51	↑
ADSL	71.02	13.06	85.89	16.50	0.004	−0.29	↓
ATIC	541.48	62.18	458.21	64.94	<0.001	0.24	↑
**Purinergic receptor**							
P2X7	353.06	86.40	428.28	91.48	0.015	−0.281	↓
P2Y2	176.50	69.64	129.19	49.99	0.006	0.450	↑
P2Y6	37.51	12.67	26.74	8.06	0.002	0.484	↑
P2Y8	3134.74	445.38	2512.10	299.43	<0.001	0.319	↑
P2Y10	389.04	81.16	485.39	62.38	<0.001	−0.319	↓

ADA, adenosine deaminase; ADSL, adenylosuccinate lyase; ATIC, bifunctional enzyme AICAR transformylase/IMP cyclohydrolase; Log2FC, Log2FoldChange.

^a^The adjusted significance level of the least significant difference was 0.05.

^b^The arrow shows the change direction of ASD group relative to TD group: ↑ means increase, ↓ means decrease.

### 3.4. Measurement of serum uric acid concentration

The Mann–Whitney *U* test was used to compare uric acid concentrations in 80 patients with ASD (median: 293.10, IQR: 94.05) and 174 children with TD (*n* = 176, median: 364.00, IQR: 110.75), with the results showing a significant difference (*p* < 0.001) (Supplementary Figure 2). Binary logistic regression and ROC analyses were performed to quantify the diagnostic performance of uric acid. Sex, age, and uric acid (odds ratio [OR], 0.979; 95% confidence interval [CI], 0.973–0.985; *P* < 0.001) were entered into the regression model, with an overall correct prediction rate of 75.2% (Supplementary Table 4). The AUC for uric acid in the validation set was 0.812 (*P* < 0.001) (Supplementary Figure 3), with a sensitivity of 82.5%, specificity of 63.8%, Youden index of 0.463, and corresponding uric acid concentration of 347.7 umol/L.

## 4. Discussion

**FIGURE 3:**
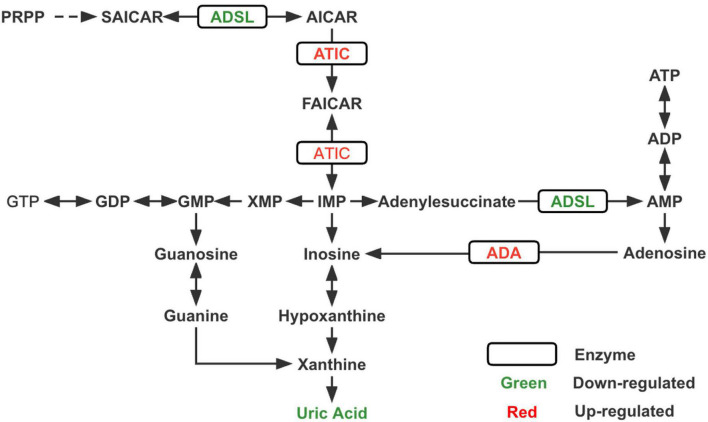
Changes in key enzymes and the end product uric acid in the purine metabolism pathway of ASD. PRPP, phosphoribosylpyrophosphate; SAICAR, succinylaminoimidazole carboxamide ribotide; AICAR, aminoimidazole carboxamide ribotide; FAICAR, formyloaminoimidazole carboxamide ribotide; IMP, inosine monophosphate; AMP, adenine monophosphate; ADP, adenosine diphosphate; ATP, adenosine triphosphate; XMP, xanthine monophosphate; GMP, guanine monophosphate; GDP, guanine diphosphate; GTP, guanine triphosphate; ADSL, adenylosuccinate lyase; ATIC, bifunctional enzyme AICAR transformylase/IMP cyclohydrolase; ADA, adenosine deaminase.

To the best of our knowledge, this is the first study to utilize metabolomics complemented with transcriptomics in ASD. Through metabolomics analysis, we verified that the purine metabolic pathway was significantly altered in patients with ASDs and found that uric acid was one of the most significant differential metabolites between ASDs and TDs. RNA-seq analysis revealed significant differences in the transcriptional expression of several key enzymes of purine metabolism (ADA, ADSL, and ATIC) (Figure 3; Supplementary Figure 4) and purinergic signaling receptor genes (P2Y2, P2Y6, P2Y8, P2X7, and P2Y10) between the two groups.

In this study, uric acid, an antioxidant, was reduced in the blood of patients with ASD. Similarly in the Chinese Han population, Wang et al. (2016) also found reduced serum uric acid levels in children with ASD; however, when logistic regression analysis was performed, uric acid became less significant. The metabolomic results of that study also showed significant changes in docosahexaenoic acid and sphingosine 1-phosphate, which finding was not obtained in the present study. The reasons for this discrepancy may be due to differences in technical or analytical methodologies, and the issue of dietary control prior to sample collection may also have a different impact on the results. In addition, there is inconsistent metabolism of uric acid levels in the urine of children with ASD. Multiple metabolomic studies have identified a significant decrease in uric acid levels in the urine of patients with ASD. In contrast, Page and Coleman (2000) observed hyperuricemic metabolism in some patients with autism subtypes, which was attributed to increased *de novo* purine synthesis in these patients. We speculate that there are two possible reasons for the inconsistent metabolism of uric acid. First, the metabolism varies between ASD subtypes; second, the metabolism can be affected by drugs, such as an increase in serum uric acid in ASD patients treated with risperidone (Vanwong et al., 2016).

According to our transcriptomic results, ADSL expression was reduced, whereas ADA and ATIC expression were increased, and these three enzymes were closely associated with *de novo* purine synthesis (Toth and Yeates, 2000; Cristalli et al., 2001; Boutchueng-Djidjou et al., 2015). Previous studies (Stubbs et al., 1982; Marie et al., 2004) related to neurodevelopmental disorders have shown reduced concentrations or deficiencies of ADA and ATIC, while very few cases of elevated expression levels have been reported. ADSL deficiency leads to autism-related features, which have been consistently reported in studies on inborn errors of metabolism (IEM) associated with ASD (Jaeken and Van den Berghe, 1984; Jaeken et al., 1988; Cohen et al., 2005). Accordingly, we speculate that the reduced uric acid concentration in ASD may be due to an imbalance in purine metabolism, the most critical reason for which is probably the decreased expression of upstream ADSL. However, a noteworthy point is that innate errors associated with purine synthesis and metabolism may arise from mutations in specific proteins, which interfere with the function of various key enzymes to different degrees and affect their activity (Dewulf et al., 2022). Therefore, measuring the expression of enzymes alone is not sufficient, but the activity of enzymes should also be considered.

Oxidative stress has been implicated as one of the pathophysiological mechanisms underlying ASD, which may be caused by an imbalance between endogenous/exogenous prooxidant generation and antioxidant defense mechanisms against reactive oxygen species (ROS) in children with ASD (Kern and Jones, 2006; Manivasagam et al., 2020). Considering that blood uric acid can reflect uric acid levels in the cerebrospinal fluid (Bowman et al., 2010), this suggests that the decrease in uric acid may lead to the CSN of ASD patients experiencing more oxidative stress, which affects the neurodevelopment process. We further compared the blood uric acid levels in a larger sample of ASD and TD children, and the results were significantly different. Moreover, uric acid showed excellent diagnostic performance in both the metabolomic and validation sample sets. Therefore, we believe that uric acid can be used as a reliable biomarker for ASD, which is not only easy to detect but also cost-effective.

Additionally, this study found that the expression of the P2X7 receptor was lower in children with ASD. Naviaux et al. (2013) found that the expression of the P2X7 receptor decreased in the ASD mouse model, which returned to normal after treatment with antipurinergic drugs. Under pathological conditions, the purinergic receptor is upregulated or downregulated, which regulates the occurrence and development of CNS inflammatory responses in a complex manner (Huang et al., 2019). Among the P2X subtypes, P2X7 plays a key role in the pathophysiology of CNS disorders and mediates the strongest evidence of neuroinflammation (Lister et al., 2007; Sperlágh and Illes, 2014). Microglia are key regulators of the neuroinflammatory response, and when microglia-dependent P2X7 receptors are activated, a range of pro-inflammatory cytokines such as IL-1β are released (Monif et al., 2009; Matta et al., 2019). Thus, our results may provide preliminary research clues regarding the mechanism of neuroinflammatory action in ASD.

There are several limitations in this study that deserve mention. First, our omics sample size was relatively small due to the unique nature of children with ASD, where most children did not cooperate well during blood collection, making it difficult to obtain blood samples. Second, there was an imbalance in the ratio of males to females in this study. This is because in the ASD population, the prevalence is higher in men than in women (Maenner et al., 2021), and therefore we collected a higher proportion of male samples. Third, only the expression levels of enzymes related to purine metabolism were examined in this study. However, in fact, the expression status of enzyme activity is also important for the disease, and attention should be paid to measuring enzyme activity in future studies as well. Fourth, the food intake of the children prior to sample collection was not controlled in this study, while diet plays an important role in metabolomics studies. We included transcriptomics to increase the reliability of the metabolomic results because the diet does not affect transcript levels. Nevertheless, our study examined changes in the blood uric acid and purine metabolic pathways in patients with ASD at the metabolic and transcriptional levels, providing broader insights for future studies. If follow-up studies can combine with genomics, it will be more convincing to verify changes in purine metabolism at the genetic level.

## 5. Conclusion

In summary, blood uric acid levels were significantly lower in children with ASD, and there were differences in the transcript levels of multiple purine metabolism-related genes between children with ASD and TD children. The results of this study suggest that serum uric acid may be used as a biomarker for objective diagnosis of a subtype of ASD, which may provide a valuable reference for more accurate treatment of ASD in the future.

## Data availability statement

The datasets presented in this article are not readily available because the original data of this study must be filed and approved by the Ministry of Science and Technology of the People’s Republic of China before it can be opened to the public. The data presented in this study will be deposited in the MetaboLights www.ebi.ac.uk/metabolights/MTBLS6926 and Sequence Read Archive (SRA) http://www.ncbi.nlm.nih.gov/bioproject/925652 repositories, accession numbers MTBLS6926 and PRJNA925652, respectively.

## Ethics statement

The studies involving human participants were reviewed and approved by the Ethics Committee of the Second Xiangya Hospital of Central South University. Written informed consent to participate in this study was provided by the participants’ legal guardian/next of kin.

## Author contributions

JO and YS designed research, acquired funding, and revised the manuscript. SD performed research, analyzed data, and wrote the manuscript. JL performed research and analyzed transcriptomics data. YH performed the research. XL provided comments on the study design and performed research. All authors contributed to the article and approved the submitted version.

## Footnotes

1
https://www.mzcloud.org/

2
http://www.chemspider.com/

3
https://www.metaboanalyst.ca/
