# Supplementary material for: Purine signaling pathway dysfunction in autism spectrum disorders: Evidence from multiple omics data
Source: Front Mol Neurosci. 2023 Feb 3;16:1089871. doi: 10.3389/fnmol.2023.1089871 (PMC9935591; doi:10.3389/fnmol.2023.1089871)
Supplement: Supplementary file 2 [file Data_Sheet_1.docx]

Supplementary Material

# Supplementary Data

**1.1 Supplementary Methods S1**

**Untargeted Metabolomics**

**Metabolites Extraction**

Tissues (100 mg) were individually grounded with liquid nitrogen and the homogenate was resuspended with prechilled methanol and 0.1% formic acid by well vortexing. The samples were incubated on ice for 5 min and then were centrifuged at 15000 rpm, 4°C for 5 min. A some of supernatant was diluted to final concentration containing 60% methanol by LC-MS grade water. The samples were subsequently transferred to a fresh Eppendorf tube with 0.22 μm filter and then were centrifuged at 15000 g, 4°C for 10 min. Finally, the filtrate was injected into the LC-MS/MS system analysis.

**UHPLC-MS/MS Analysis**

LC-MS/MS analyses were performed using a Vanquish UHPLC system (Thermo Fisher, MA, USA) coupled with an Orbitrap Q Exactive HF-X mass spectrometer (Thermo Fisher, MA, USA). Samples were injected onto an Hyperil Gold column (100×2.1 mm, 1.9μm) using a 16-min linear gradient at a flow rate of 0.2 mL/min. The eluents for the positive polarity mode were eluent A (0.1% FA in Water) and eluent B (Methanol). The eluents for the negative polarity mode were eluent A (5 mM ammonium acetate, pH 9.0) and eluent B (Methanol). The solvent gradient was set as follows: 2% B, 1.5 min; 2-100% B, 12.0 min; 100% B, 14.0 min; 100-2% B, 14.1 min; 2% B, 16 min. Q Exactive HF-X mass spectrometer was operated in positive/negative polarity mode with spray voltage of 3.2 kV, capillary temperature of 320°C, sheath gas flow rate of 35 arb and aux gas flow rate of 10 arb.

**1.2 Supplementary Methods S2**

**Transcriptomics study**

**Total RNA extraction from peripheral blood**

Total RNA from peripheral blood was thawed at low temperature, then 250 μl of Tris- NH4Cl (Trihydroxymethyl aminomethane, Tris, Huamei Bioengineering Co., Ltd, Wuhan, China) was added. The supernatant was discarded and 500 μl of phosphate buffer (pH=7.2) was added for suspension, followed by centrifugation at 12,000 rpm for 5 min, and the supernatant was discarded and fixed to 50 μl.

The extraction was performed according to the method of guanidinium isothiocyanate-phenol-chloroform-isopentyl alcohol (Huamei Biological Engineering Co., Ltd, Wuhan, China). To the above 50 μl of sample solution, 500 μl of the denaturing solution was added, and the ratio of this denaturing solution was 4 mol/L of guanidine isothiocyanate, 0.125 mol/L of β-mercaptoethanol (Servicebio Technology Co., Ltd, Wuhan, China), 26 mol/L of sodium citrate (Servicebio Technology Co., Ltd, Wuhan, China), and 0.5% of sodium dodecanoic (Servicebio Technology Co., Ltd, Wuhan, China).

After shaking for about 1 min using a vortex mixer, 50 ul of sodium acetate at a concentration of 2 mol/L was added sequentially, followed by 500 μl of water-saturated phenol and mixing again, and finally 100 μl of chloroform-isoamyl alcohol solution in a 24:1 ratio. The solution is then vigorously shaken and centrifuged at 12,000 rpm for 5 min, at which point the liquid is divided into two clear phases. If there is no clear division into two phases, appropriate amount of chloroform-isoamyl alcohol solution should be added to the centrifuged solution, shaken vigorously again, and centrifuged.

The RNA distributed in the upper aqueous phase was transferred to a sterile centrifuge tube, 500 μl of isopropanol was added, mixed well, and then placed in a refrigerator at - 20°C for 30 min before being removed to precipitate the RNA. Then, centrifuge at 12,000 rpm for 5 min and discard the supernatant. The precipitated fraction was washed twice with 75% ethanol (Tiangen Biochemical Technology Co., Ltd, Beijing, China) using DEPC water (Tiangen Biochemical Technology Co., Ltd, Beijing, China) and inverted at room temperature for 10 min to remove the maximum amount of ethanol from the inside of the centrifuge tube. Add DEPC water to dissolve the precipitate, vortex, and mix well to obtain the dissolved RNA sample.

**Determination of RNA purity and concentration**

In this study, RNase-free aqueous solution was used as a blank control, and the concentration and purity of total RNA solution in each sample were measured by Qubit2.0 Fluorometer and NanoPhotometer spectrophotometer (Eppendorf, Hamburg, Germany), respectively. The absorbance values at 260 nm and 280 nm of the extracted samples were used to determine the quality of the extracted samples based on the ratio of OD260/280 nm, and a value between 1.8 and 2.1 indicated that the extracted RNA was of high purity. The integrity of the sample information was measured by the RNA Integrity Number (RIN), which was determined by agarose gel electrophoresis and Agilent bioanalyzer 2100 (Agilent Technologies, CA, USA). At the end of electrophoresis, two very bright and dense ribosomal RNAs, 28S and 18S, with high molecular weight, were visible under UV transmission light. 28S/18S can be used to assess sample integrity, and RNA integrity is good when this indicator is greater than 1.5. RIN values >= 6.0 and 28S/18S >= 0.7 indicate no DNA or protein contamination.

**Transcriptome sequencing**

**Library preparation**

A total amount of 1 μg RNA per sample was used as input material for the RNA sample preparations. Briefly, mRNA was purified from total RNA using poly-T oligo-attached magnetic beads. Fragmentation was carried out using divalent cations under elevated temperature in First Strand Synthesis Reaction Buffer(5X). First strand cDNA was synthesized using random hexamer primer and M-MuLV Reverse Transcriptase (RNase H-). Second strand cDNA synthesis was subsequently performed using DNA Polymerase I and RNase H. Remaining overhangs were converted into blunt ends via exonuclease/polymerase activities. After adenylation of 3’ ends of DNA fragments, Adaptor with hairpin loop structure was ligated to prepare for hybridization. To select cDNA fragments of preferentially 370~420 bp in length, the library fragments were purified with AMPure XP system (Beckman Coulter, Beverly, USA). Then PCR was performed with Phusion High-Fidelity DNA polymerase, Universal PCR primers and Index (X) Primer. At last, PCR products were purified (AMPure XP system) and library quality was assessed on the Agilent Bioanalyzer 2100 system.

**Clustering and sequencing (Novogene Experimental Department)**

The clustering of the index-coded samples was performed on a cBot Cluster Generation System using TruSeq PE Cluster Kit v3-cBot-HS (Illumia, CA, USA) according to the manufacturer’s instructions. After cluster generation, the library preparations were sequenced on an Illumina Novaseq platform. The basic principle of sequencing is sequencing while synthesizing. After completing the library inspection and passing the qualification inspection, the different libraries were sequenced according to the requirements of effective concentration and target offline data volume, and 150bp paired end readings were generated. During the amplification of the sequenced flow cells, when the sequencing cluster extends the complementary strand, the sequencer and the computer work together to convert the fluorescence signal released from dNTP into sequencing peaks, thus obtaining the sequence information of the fragment to be detected.

**1.3 Supplementary Methods S3**

**Network analysis**

Network analysis was performed using the MetaboAnalyst 5.0 (https://www.metaboanalyst.ca/) web tool. The 66 differential metabolites and their log2FC values were entered into the Network Analysis module of the website, the Compound Name type was selected, the data was then uploaded for metabolite name review, and finally, the Metabolite-Metabolite Interaction Network analysis step was selected to obtain potential functional relationships between annotated metabolites. In the default mode of the website, the Degree cutoff is set to 2.0 and the Betweenness cutoff is set to 1.0. In the results presentation interface, the color and presentation of the images can be set according to the requirements, and most details can be viewed by zooming in and out in the interface.

# Supplementary Figures and Tables

## Supplementary Figures


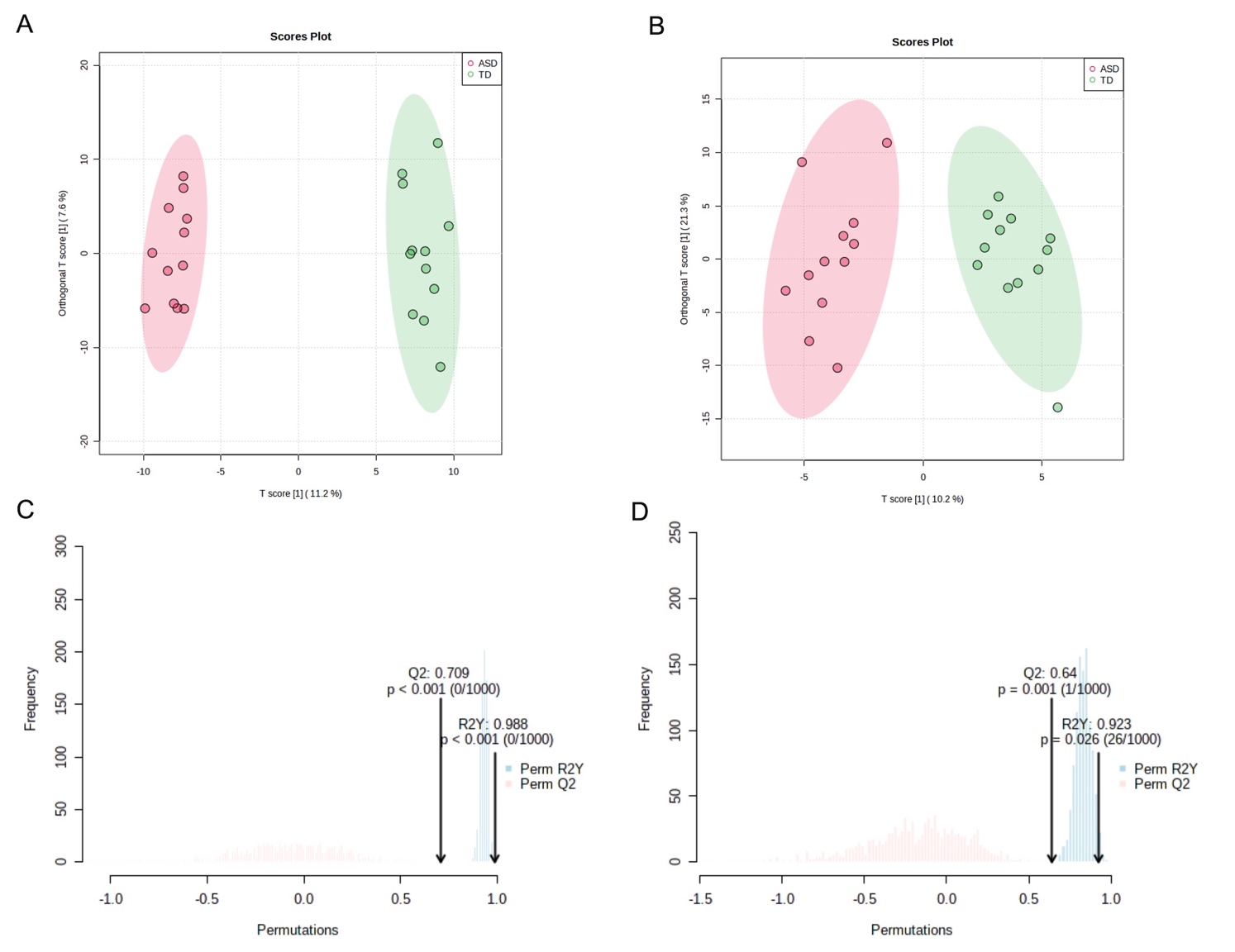


**Supplementary Figure S1.** Metabolomic analysis results of ASD group and healthy control group. (A) and (B) are OPLS-DA models of positive and negative ion metabolites, respectively; The dots in the red area represent ASD children and the dots in the green area represent healthy control. (C–D) permutation tests of OPLS-DA models for positive and negative ion metabolites through 1000 permutations; R2Y represents the interpretation rate of the model, Q2 represents the prediction rate of the model. The closer the values of R2Y and Q2 are to 1 the higher the accuracy of the model fit, and a p-value > 0.05 is significant.


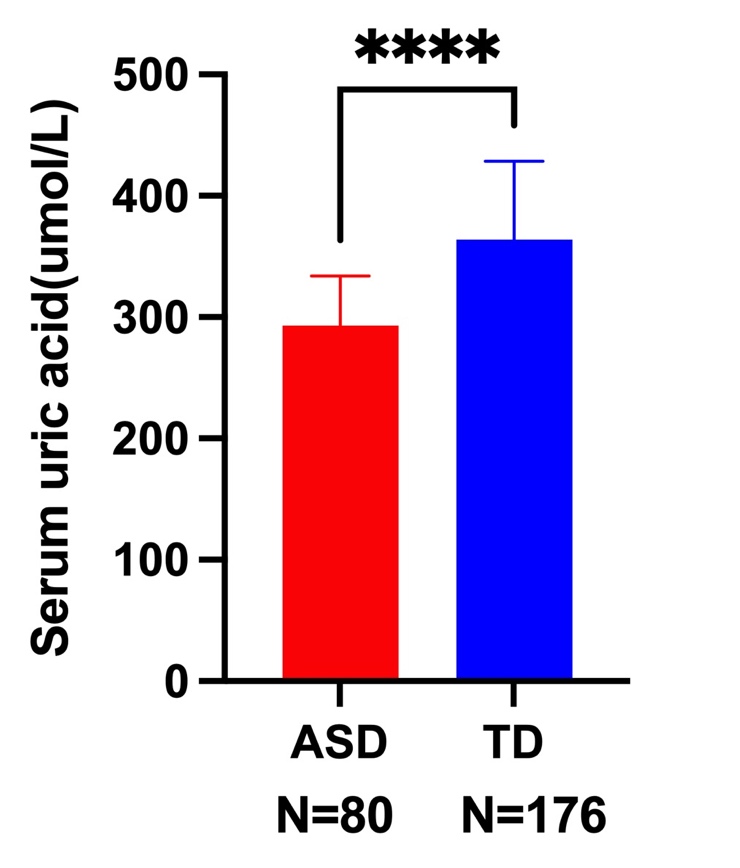


**Supplementary Figure S2.** Serum uric acid concentrations in ASD children and TD children. In the validation sample set, the ASD group had significantly lower serum uric acid levels than the TD group. ****P < 0.001 (Mann–Whitney u test).


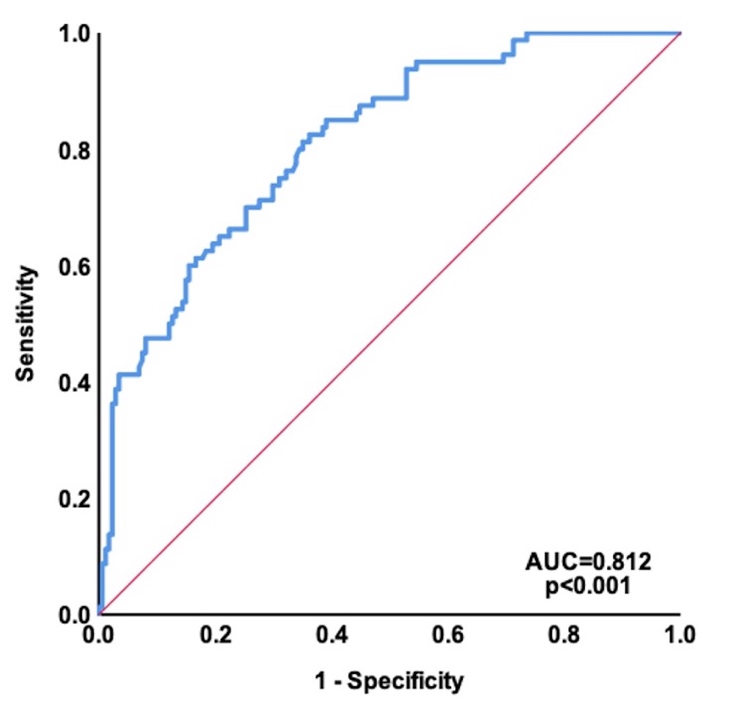


**Supplementary Figure S3.** ROC curve of uric acid for validation sample set.


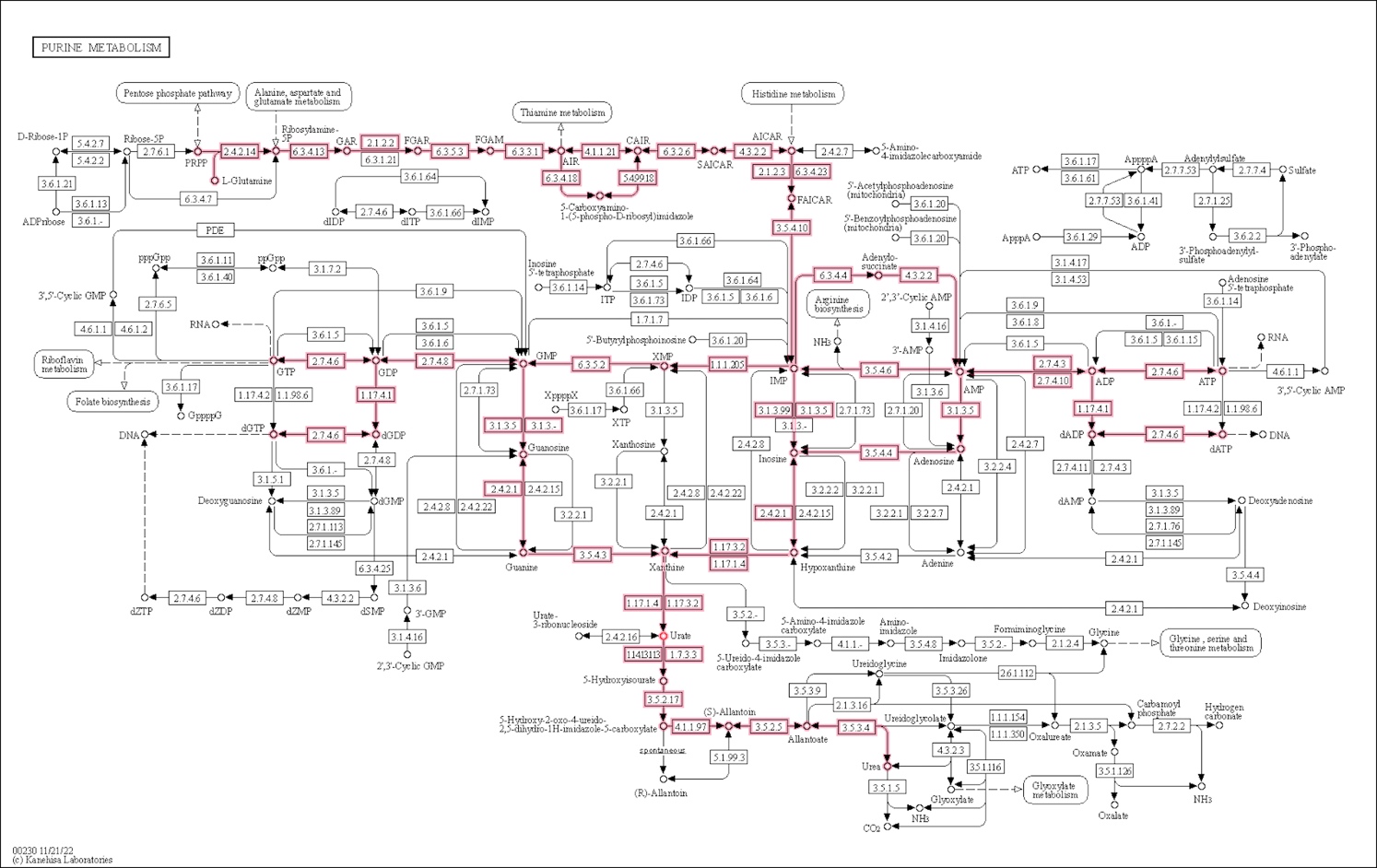


**Supplementary Figure S4.** KEGG pathway diagram of purine metabolism(map00230). Shown in red are the key metabolites and key enzymes from the starting point (PRPP) to the end point (Urea) of purine metabolism.

## Supplementary Tables

**Supplementary Table S1.** Pathway enrichment results in metabolite-metabolite network analysis.

| **Pathway** | **Total^1^** | **Expected** | **Hits^2^** | **P. Value^3^** | **Topology^4^** | **PVal.Z** | **Topo.Z** |
| --- | --- | --- | --- | --- | --- | --- | --- |
| Purine metabolism | 65 | 2.47 | 15 | 4.04E-09^***^ | 0.875 | 6.88 | 4.21 |
| Steroid hormone biosynthesis | 85 | 3.23 | 14 | 1.36E-06^***^ | 0.702 | 4.7 | 3.28 |
| Pyrimidine metabolism | 39 | 1.48 | 6 | 0.00287^**^ | 0.526 | 1.84 | 2.32 |
| Glyoxylate and dicarboxylate metabolism | 32 | 1.22 | 4 | 0.0306^*^ | 0.258 | 0.95 | 0.865 |
| Pantothenate and CoA biosynthesis | 19 | 0.723 | 3 | 0.0328^*^ | 0.278 | 0.924 | 0.972 |
| Glycine, serine and threonine metabolism | 33 | 1.26 | 4 | 0.0339^*^ | 0.188 | 0.912 | 0.482 |
| Pentose phosphate pathway | 22 | 0.837 | 3 | 0.0482^*^ | 0.143 | 0.781 | 0.24 |
| Glutathione metabolism | 28 | 1.07 | 3 | 0.0873 | 0.593 | 0.558 | 2.68 |
| Riboflavin metabolism | 4 | 0.152 | 1 | 0.144 | 0.667 | 0.371 | 3.08 |
| beta-Alanine metabolism | 21 | 0.799 | 2 | 0.189 | 0.65 | 0.27 | 2.99 |

^1^The total number of compounds in the pathway; ^2^The actually matched number from the uploaded data; ^3*^p<0.05, ^**^p<0.01, ^***^p<0.001; ^4^For comparison among different pathways, the node importance values calculated from centrality measures are further normalized by the sum of the importance of the pathway. Therefore, the total/maximum importance of each pathway is 1; the importance measure of each metabolite node is actually the percentage of the total pathway importance, and the pathway impact is the cumulative percentage from the matched metabolite nodes.

**Supplementary Table S2.** Receiver operator characteristic (ROC) curve analysis of significant metabolites and genes for the diagnosis of ASD.

| **Metabolite and Gene^1^** | **AUC (95%CI)^2^** | **Sensitivity (%)** | **Specificity (%)** | **Maximum of Youden index^3^** | **P value^4^** |
| --- | --- | --- | --- | --- | --- |
| Metabolite |  |  |  |  |  |
| Uric acid | 0.958(0.882-1.000) | 91.70 | 91.70 | 0.834 | <0.001^***^ |
| 3,3',4'5-tetrahydroxystilbene | 0.931(0.833-1.000) | 75.00 | 100.00 | 0.75 | <0.001^***^ |
| Dihydrotestosterone | 0.882(0.740-1.000) | 91.70 | 83.30 | 0.750 | 0.001^**^ |
| Allantoic acid | 0.854(0.704-1.000) | 91.70 | 66.70 | 0.584 | 0.003^**^ |
| Bilirubin | 0.847(0.677-1.000) | 91.70 | 75.00 | 0.667 | 0.004^**^ |
| D-Pantothenic Acid | 0.833(0.672-0.995) | 91.70 | 66.70 | 0.584 | 0.006^**^ |
| purine metabolism-related genes |  |  |  |  |  |
| ADA | 0.853(0.747-0.960) | 54.20 | 100 | 0.542 | <0.001^***^ |
| ATIC | 0.819(0.689-0.950) | 83.30 | 80.95 | 0.643 | <0.001^***^ |
| ADSL | 0.778(0.638-0.917) | 85.70 | 66.67 | 0.524 | 0.001^**^ |
| purinergic receptor genes |  |  |  |  |  |
| P2Y2 | 0.772(0.632-0.912) | 66.70 | 85.71 | 0.524 | 0.002^**^ |
| P2Y6 | 0.754(0.610-0.898) | 75.00 | 76.19 | 0.512 | 0.004^**^ |
| P2Y8 | 0.889(0.792-0.986) | 83.30 | 85.71 | 0.690 | <0.001^***^ |
| P2X7 | 0.760(0.613-0.907) | 71.40 | 16.70 | 0.548 | 0.003^**^ |
| P2Y10 | 0.829(0.712-0.947) | 87.50 | 42.86 | 0.304 | <0.001^***^ |
| P2Y4 | 0.667(0.507-0.827) | 33.30 | 85.71 | 0.190 | 0.056 |
| P2Y14 | 0.647(0.481-0.812) | 25.00 | 95.24 | 0.202 | 0.092 |

^1^DPYSL-2, dihydropyrimidinase-like 2; ADA, adenosine deaminase; ATIC, bifunctional enzyme AICAR transformylase/IMP cyclohydrolase; ADSL, adenylosuccinate lyase;^2^AUC = area under the curve; ^3^Sensitivity+specificity-1; ^4**^p<0.01, ^***^p<0.001.

**Supplementary Table S3.** Comparison of normalized counts of gene expression in ASD and TD children.

| **Gene Name^1^** | **ASD(n=24)** | |  | **TD(n=21)** | |  | **Between-group difference** | | |
| --- | --- | --- | --- | --- | --- | --- | --- | --- | --- |
|  | **Mean** | **SD** |  | **Mean** | **SD** |  | **p-adj^2^** | **Log2FoldChange** | **Trend** |
| purine metabolism-related genes |  |  |  |  |  |  |  |  |  |
| PRPS1 | 582.17 | 69.04 |  | 568.87 | 77.16 |  | 0.645 | 0.03 | ↑ |
| ADA | 456.09 | 79.24 |  | 353.47 | 59.63 |  | <0.001^***^ | 0.51 | ↑ |
| ADSL | 71.02 | 13.06 |  | 85.89 | 16.50 |  | 0.004^**^ | -0.29 | ↓ |
| ATIC | 541.48 | 62.18 |  | 458.21 | 64.94 |  | <0.001^***^ | 0.24 | ↑ |
| HPRT | 151.57 | 24.99 |  | 148.84 | 24.63 |  | 0.778 | 0.08 | ↑ |
| purinergic receptor |  |  |  |  |  |  |  |  |  |
| A1 | 6.52 | 5.66 |  | 6.23 | 5.16 |  | 0.915 | 0.052 | ↑ |
| A2A | 30.81 | 8.11 |  | 30.69 | 9.22 |  | 0.889 | 0.024 | ↑ |
| A2B | 37.49 | 12.59 |  | 37.53 | 13.51 |  | 0.968 | -0.009 | ↓ |
| A3 | 65.94 | 37.62 |  | 55.50 | 41.49 |  | 0.420 | 0.248 | ↑ |
| P2X1 | 1066.86 | 239.60 |  | 1005.66 | 383.21 |  | 0.599 | 0.086 | ↑ |
| P2X4 | 473.88 | 86.65 |  | 468.66 | 58.34 |  | 0.874 | 0.015 | ↑ |
| P2X5 | 601.06 | 162.74 |  | 552.28 | 96.53 |  | 0.350 | 0.122 | ↑ |
| P2X6 | 13.07 | 7.32 |  | 12.27 | 5.66 |  | 0.769 | 0.092 | ↑ |
| P2X7 | 353.06 | 86.40 |  | 428.28 | 91.48 |  | 0.015^*^ | -0.281 | ↓ |
| P2Y1 | 27.06 | 7.87 |  | 27.50 | 7.89 |  | 0.982 | -0.004 | ↓ |
| P2Y2 | 176.50 | 69.64 |  | 129.19 | 49.99 |  | 0.006^**^ | 0.450 | ↑ |
| P2Y4 | 3.04 | 1.98 |  | 4.32 | 2.10 |  | 0.156 | -0.478 | ↓ |
| P2Y6 | 37.51 | 12.67 |  | 26.74 | 8.06 |  | 0.002^**^ | 0.484 | ↑ |
| P2Y8 | 3134.74 | 445.38 |  | 2512.10 | 299.43 |  | <0.001^***^ | 0.319 | ↑ |
| P2Y10 | 389.04 | 81.16 |  | 485.39 | 62.38 |  | <0.001^***^ | -0.319 | ↓ |
| P2Y11 | 183.41 | 31.31 |  | 191.79 | 41.86 |  | 0.565 | -0.064 | ↓ |
| P2Y12 | 20.25 | 9.86 |  | 18.81 | 17.38 |  | 0.759 | 0.109 | ↑ |
| P2Y13 | 3097.34 | 795.85 |  | 3261.72 | 1037.59 |  | 0.636 | -0.075 | ↓ |
| P2Y14 | 161.53 | 61.18 |  | 189.13 | 79.82 |  | 0.216 | -0.229 | ↓ |

^1^PRPS, phosphoribosyl pyrophosphate synthase; ADA, adenosine deaminase; ADSL, adenylosuccinate lyase; ATIC, bifunctional enzyme AICAR transformylase/IMP cyclohydrolase; HPRT, hypoxanthine guanine phosphoribosyltransferase; DPYD, dihydropyrimidine dehydrogenase; DPYSL-2, dihydropyrimidinase-like 2; ^2^The adjusted significance level of the least significant difference was 0.05; ^*^p<0.5, ^**^p<0.01, ^***^p<0.001.

**Supplementary Table S4.** Binary logistic regression models for validation groups.

| **Factor** | **B** | **Wald χ^2^** | **P value^1^** | **OR (95%CI)** |
| --- | --- | --- | --- | --- |
| Sex | -0.719 | 1.476 | 0.224 | 0.487 (0.153-1.554) |
| Age | 0.165 | 10.536 | 0.001^**^ | 1.179 (1.068-1.303) |
| Uric acid | -0.022 | 52.023 | <0.001^***^ | 0.978 (0.973-0.984) |

^1**^p<0.01, ^***^p<0.001.
